# Fluctuations in the Number of Stores by Industry During the COVID-19 Pandemic Based on Japanese Phone Book Entries

**DOI:** 10.1007/s12626-022-00120-0

**Published:** 2022-10-09

**Authors:** Saki Saito, Mariko I. Ito, Takaaki Ohnishi

**Affiliations:** 1 Graduate School of Artificial Intelligence and Science, Rikkyo University, Tokyo, Japan; 2 Institute of Industrial Science, The University of Tokyo, Tokyo, Japan; 3 Graduate School of Artificial Intelligence and Science, Rikkyo University, The Canon Institute for Global Studies, Tokyo, Japan

**Keywords:** Telephone directory data, COVID-19 outbreak, Stores and facilities, Bankruptcies

## Abstract

Currently in Japan, summaries of the number of bankruptcies due to the spread of COVID-19 can only be obtained from surveys conducted by a few research firms targeting particular companies. In this study, we used Japanese telephone directory data containing detailed information on the location and industrial category of stores/facilities nationwide in an effort to infer the influence of COVID-19 on businesses in Japan. We analyzed the temporal change in the number of stores before and after the COVID-19 outbreak. Among other findings, the analysis revealed that the number of travel agencies and facilities offering karaoke and other forms of entertainment declined significantly after the outbreak in some prefectures, with the largest declines in Ibaraki, Osaka, and Hyogo prefectures, and a relatively small decline in Tochigi prefecture. Among the stores and facilities categorized as restaurants and travel-related services, the decline was particularly significant in urban areas such as Tokyo and Osaka prefectures.

## Introduction

Economic activities can be affected by various exogenous events, including health crises such as the worldwide outbreak of the novel coronavirus disease (COVID-19) [1–4]. The first case of COVID-19 in Japan was reported in January 2020, followed by several large outbreaks with clusters of the infection. As of April 2022, the cumulative number of cases of COVID-19 in Japan has exceeded 7.6 million. In its efforts to combat the spread of the virus, the Japanese government has declared a state of emergency four times: from April to May 2020, January to March 2021, April to June 2021, and July to September 2021. In each case, the people were asked to refrain from going out after 8 p.m. and to limit their social interactions. Unsurprisingly, these measures have had a serious impact on economic activities throughout the country.

It is estimated that 700 Japanese firms with total indebtedness greater than 10 million yen went bankrupt due to the pandemic in 2020, followed by 1700 more firms in 2021, according to Teikoku Databank, Ltd [5]. Measures against such widespread company bankruptcies are required for economic stability [6]. Currently, precise information on bankruptcies in Japan is mostly limited to the company information provided by domestic research firms such as Teikoku Databank and Tokyo Shoko Research [7]. Little if any detailed information regarding the changes in the number of stores/facilities in Japan during the COVID-19 pandemic appears to be available

In this study, we used telephone directory data containing not only phone numbers but also information on the location and industrial category of each store/facility in order to estimate the changes in the number of stores/facilities by industry before and after the COVID-19 outbreak. Using such data, we were able to count the number of records for stores/facilities in a certain prefecture or in a certain industrial category. We approximated the number of stores/facilities using the number of records in the data and calculated the value at each of 12 time points. By evaluating the temporal change in the number or change rate of the number, we were able to identify areas where the number of stores/facilities in various industrial categories decreased significantly after the outbreak.

The rest of this paper is organized as follows. In Sect. 2, we explain the data and the method. We show the results on the change in the number of stores/facilities in Sect. 3, followed by a discussion in Sect. 4.

## Method

To conduct our study, we analyzed the NTT Hello Page phone book provided by Eins Co., Ltd. (https://teldata.jp/). This dataset is updated every few months. For each store or facility, the telephone directory data record includes the store or facility’s telephone number, name, zip code, prefecture, city, address, and industrial category. Multiple industrial categories are sometimes assigned to a single store or facility. Thus, the dataset provides an approximation of the number of existing stores and facilities both temporally and geographically. Each industry code is represented by an alphabetical character, and each industrial category is further classified into detailed industrial categories, each of which has a specific code called a detailed industry code, denoted by a three-digit number. For example, the detailed industry code A100 denotes a “dental clinic” in the medical industrial category.

**Table 1:** Industry codes

Industry code	Category
A	Medical care
B	Restaurants
C	Manufacturing
D	Cars and motorcycles
E	Transportation
F	Construction
G	Professional services, various diagnostics and consulting
H	Real estate business
I	Wholesale
J	Retail (food and food ingredients)
K	Retail (lifestyle and goods)
L	Retail (beauty and fashion)
M	Housing (equipment and air conditioning)
N	Housing (exterior)
O	Housing (interior)
P	Services (beauty and fashion)
Q	Services (living)
R	Services (others)
S	Sports and leisure
T	Travel
U	Education and culture
V	Weddings, funerals and events
W	Public institution
X	Finance, publishing and infrastructure
Y	Primary industry

In the directory data analyzed, there were 25 industry codes and 332 detailed industry codes, as shown in Table 1. We analyzed the data at 12 time points, from September 2018 to May 2021. Each time point *t* is listed in Table 2. The number of stores at time *t* is denoted as $N_t$. Here, each time point *t* at which the information is aggregated in the data is not evenly distributed, whereas the data are provided regularly. Approximately five million total records are represented at each time point (Table 2). We can observe a trend before the outbreak of COVID-19 in which the number of stores/facilities recorded in the data generally decreases. However, it should be noted that although such a decrease in the total number of records can be observed, this does not necessarily mean an increase in the number of stores that have closed, since the telephone directory dataset provides an incomplete picture of the number of existing stores/facilities. For example, it does not include stores/facilities that are not registered in the telephone directory data or those without a landline telephone. However, if there is a significant difference in the number of phone book entries before and after the COVID-19 outbreak, it can be expected that this difference reflects, at least to some extent, the impact of the outbreak.

**Table 2:** Number of stores/facilities recorded in the telephone directory data for each time point

*t*	Month	Number of stores/facilities
1	September 2018	5,148,294
2	December 2018	5,099,148
3	April 2019	5,052,948
4	July 2019	5,020,870
5	October 2019	4,979,354
6	January 2020	4,946,499
7	May 2020	4,902,625
8	August 2020	4,878,092
9	October 2020	4,849,242
10	December 2020	4,821,688
11	March 2021	4,767,569
12	May 2021	4,730,553

## Results

### Temporal Changes in the Number of Stores

**Fig. 1:**
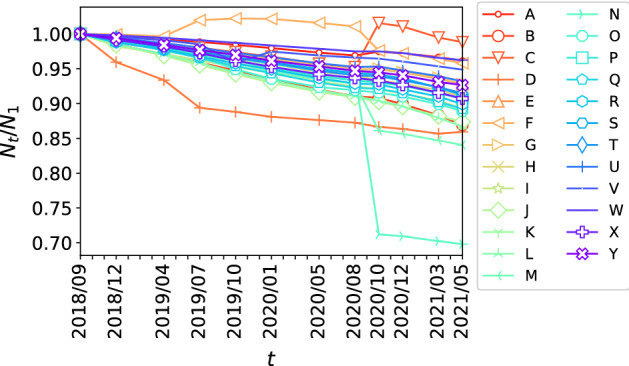
Changes in the number of stores since September 2018 ($t=1$), $N_t/N_1$. Different symbols represent different industry codes

We first consider the temporal changes in the number of stores/facilities nationwide by industry. Figure 1 shows the changes in the number of stores since September 2018 (*t*=1), $N_t/N_1$. As can be seen here, there is a continuous decline in the number of stores in the phone book over time. As previously noted, however, a decrease in $N_t/N_1$ does not directly imply an increase in the number of closed stores. Additionally, temporal changes in $N_t/N_1$ can be affected by the method used to edit the data. Here, the criteria for assigning industry codes to each store/facility or the classification of categories appeared to change at times during the analyzed period. In fact, there were several dramatic changes that led to changes in the registered industry code for many of the stores/facilities in certain industries. A significant decrease or increase in $N_t/N_1$ can be seen for industry codes C (manufacturing), D (cars and motorcycles), F (construction), M (Housing (equipment and air conditioning)), and N (housing (exterior)) in Fig. 1. For the other categories, $N_t/N_1$ decreases at a relatively constant rate. We limit our analysis to only those categories where $N_t$ and $N_1$ can be regarded as being edited under similar biases, so that the ratio $N_t/N_1$ will not be affected by bias in the data.

**Fig. 2:**
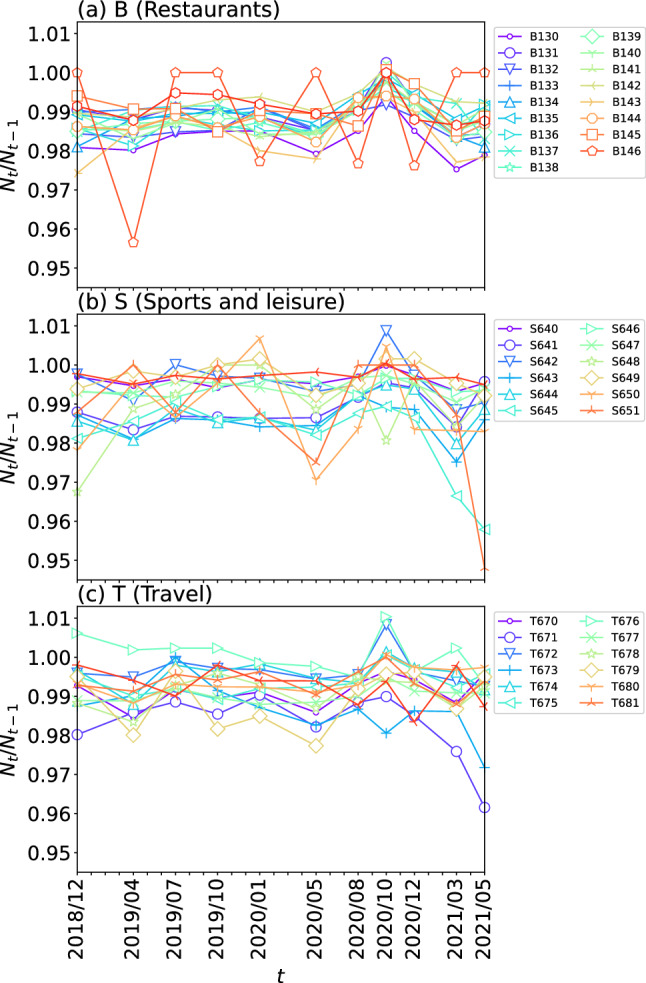
Temporal changes in the number of stores/facilities $N_t/N_{t-1}$ for each detailed industry code in the categories of (**a**) restaurants, (**b**) sports and leisure, and (**c**) travel. Different symbols represent different detailed industries

We subsequently investigated the temporal changes in the number of stores/facilities for several detailed codes. Figure 2 shows the ratio $N_t/N_{t-1}$ of the number of stores at time *t* to that at time $t-1$, indicating the extent to which the number of stores/facilities temporally increased or decreased for each detailed industry code, in the categories of restaurants (B), sports and leisure (S), and travel (T). Interestingly, the value of $N_t/N_{t-1}$ was relatively high, whereas it was generally less than 1, for many detailed industry codes in October 2020. The indication is that the decrease in the number of stores/facilities belonging to these categories was temporarily suppressed in October 2020, even though this was after the outbreak of COVID-19. In contrast, the ratio $N_t/N_{t-1}$ rapidly declined from March to May 2021 for some detailed industry codes in S and T. In category S (sports and leisure), detailed industry codes S645 (“karaoke, bowling, game centers, discos, etc.”) and S651 (“various halls”) showed large decreases in the values of $N_t/N_{t-1}$. In category T (travel), $N_t/N_{t-1}$ of T671 (“travel agency”) and T673 (“lodging reservation information”) also decreased greatly during this period. Among these subcategories showing rapid decline, the number of records for S645 and T671 was sufficiently large. Therefore, in the following, we investigate the change in the number of stores/facilities in greater detail for these two detailed industry codes.

**Table 3:** Prefectures of Japan

#	Prefecture	#	Prefecture	#	Prefecture	#	Prefecture
1	Hokkaido	13	Tokyo	25	Shiga	37	Kagawa
2	Aomori	14	Kanagawa	26	Kyoto	38	Ehime
3	Iwate	15	Niigata	27	Osaka	39	Kochi
4	Miyagi	16	Toyama	28	Hyogo	40	Fukuoka
5	Akita	17	Ishikawa	29	Nara	41	Miyazaki
6	Yamagata	18	Fukui	30	Wakayama	42	Nagasaki
7	Fukushima	19	Yamanashi	31	Tottori	43	Kumamoto
8	Ibaraki	20	Nagano	32	Shimane	44	Kagoshima
9	Tochigi	21	Gifu	33	Okayama	45	Saga
10	Gunma	22	Shizuoka	34	Hiroshima	46	Oita
11	Saitama	23	Aichi	35	Yamaguchi	47	Okinawa
12	Chiba	24	Mie	36	Tokushima		

**Fig. 3:**
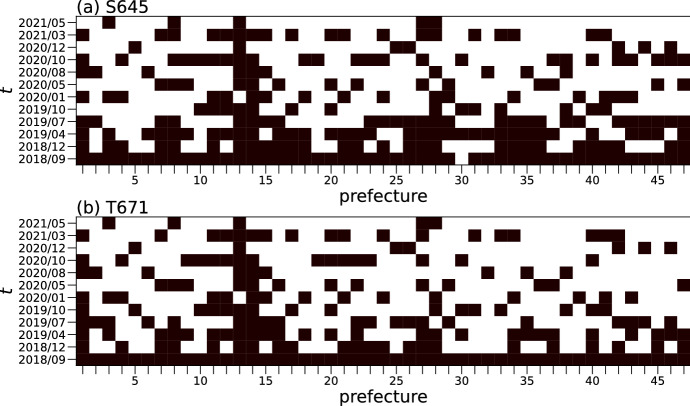
Updates of the number of stores/facilities in the telephone directory data. For each panel, a cell for a time *t* and prefecture is filled in if the number of stores in data $N_t$ has changed from that of the previous time point, data $N_{t-1}$, for detailed industry codes (**a**) S645 and (**b**) T671. All cells for September 2018 ($t=1$) are filled in black; the prefectures are numbered as shown in Table 3

Before examining the temporal changes in the numbers of stores/facilities in S645 and T671, we confirmed that the data for these detailed industry codes had been updated in each prefecture. In Fig. 3, for each detailed code, a cell for time *t* and for a certain prefecture is colored in black if the number of stores in the data $N_t$ for the focal detailed industry and prefecture had changed from that of the previous time, $N_{t-1}$. Thus, the heatmap highlights the cells in which the data have been definitely updated. We also colored all cells for September 2018 ($t=1$) in black. In this figure, the prefectures are numbered as shown in Table 3. Figure 3 shows that the data for each detailed industry code and prefecture were updated at least twice, including September 2018, although it was irregular, during the analyzed period. Furthermore, since April 2020, when the state of emergency was declared for the first time in Japan, the data were updated at least once for all prefectures, excluding Fukui, Kochi, and Kumamoto.

**Fig. 4:**
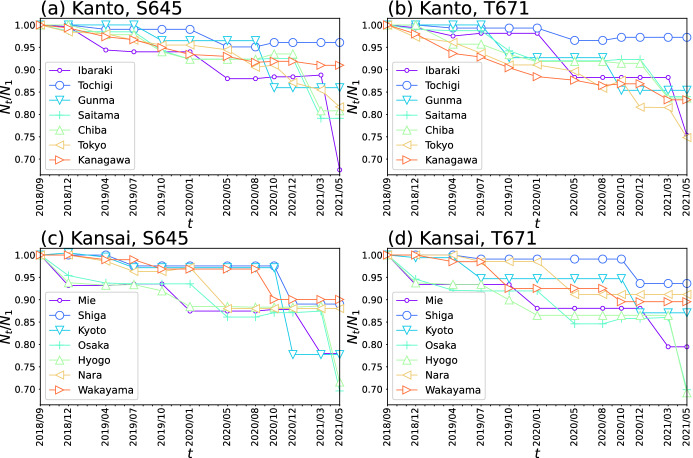
Temporal changes in the number of stores/facilities $N_t/N_{t-1}$ in each prefecture (**a**) in the Kanto region for detailed industry code S645, (**b**) in the Kanto region for T671, (**c**) in Kansai region for S645, and (**d**) in Kansai region for T671. Different markers represent different prefectures

We then investigated the change in the number of stores/facilities by prefecture for those with detailed industry codes S645 and T671. Figure 4 shows $N_t/N_{t-1}$ for all prefectures and detailed industry codes S645 (“karaoke, bowling, game centers, discos, etc.”) and T671 (“travel agency”). Here, $N_t$ denotes the number of stores/facilities with a specific detailed industry code in a prefecture at time *t*. Whether the number of stores has significantly decreased or the decrease has been controlled depends on the prefecture. Regarding S645, the values of $N_t/N_{t-1}$ dramatically decreased after March 2021 in Ibaraki, Osaka, and Hyogo prefectures. For T671, large decreases in $N_t/N_{t-1}$ after March 2021 were also observed in Ibaraki, Tokyo, Hyogo, and Osaka prefectures. However, the decreases in $N_t/N_{t-1}$ were suppressed in Tochigi and Kanagawa prefectures for S645, and in Tochigi, Nagano, and Miyazaki prefectures for T671.

### Comparison of Growth Rates for the Number of Stores/Facilities Before and After the Outbreak

**Fig. 5:**
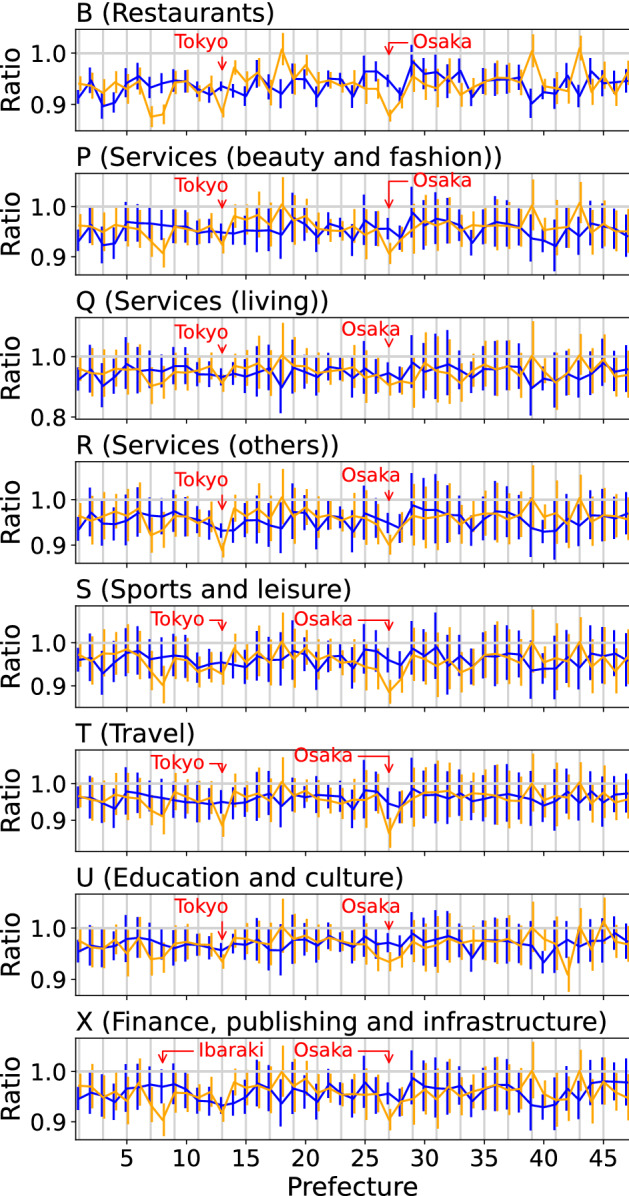
Rate of increase in the number of stores/facilities after and before the outbreak of COVID-19. Ratios $N_6/N_1$ and $N_{12}/N_6$ are shown in blue and orange, respectively, for each prefecture. Each panel shows the result for each industry, whose code is exhibited on the upper left of the panel. An error bar with a range of $\left[ (N_6-\sqrt{N_6})/(N_1+\sqrt{N_1}), (N_6+\sqrt{N_6})/(N_1-\sqrt{N_1})\right]$ is demonstrated for $N_6/N_1$. The error bar for $N_{12}/N_6$ is given in a manner similar to that for $N_6/N_1$. Prefectures are numbered as shown in Table 3

Next, we compared the rate of increase in the number of stores after the outbreak of COVID-19 to the rate before the outbreak. We calculated the ratios $N_6/N_1$ and $N_{12}/N_6$, which represent the growth rates of $N_t$ before and after the outbreak of COVID-19; $t=1,~6$, and 12 correspond to September 2018, January 2020, and May 2021, respectively. Figure 5 shows $N_6/N_1$ in blue and $N_{12}/N_6$ in orange for all prefectures and for 8 industry codes: B (restaurants), P (services (beauty and fashion)), Q (services (living)), R (various services such as advertising, telephone, and garbage collection), S (sports and leisure), T (travel), U (education and culture), and X (Finance, publishing and infrastructure). With regard to the error bars of $N_6/N_1$ and $N_{12}/N_6$, we assume that the true number of stores at time *t* follows a Poisson distribution with mean $N_t$, which was shown in the phone book data. We show an error bar for $N_6/N_1$ with a range of $\left[ (N_6-\sqrt{N_6})/(N_1+\sqrt{N_1}), (N_6+\sqrt{N_6})/(N_1-\sqrt{N_1})\right]$. The error bar for $N_{12}/N_6$ was calculated in a manner similar to that for $N_6/N_1$. If $N_{12}/N_6$ is greater (smaller) than $N_6/N_1$, we can infer the following situations: a decrease in the number of stores/facilities was suppressed (exaggerated) or a trend where a decrease (increase) in the number of stores/facilities turned to an increase (decline) became more prominent after the outbreak compared to that before it.

For industry code B (restaurants), Tokyo and Osaka prefectures had much smaller post-outbreak growth rates $N_{12}/N_6$ compared with $N_6/N_1$, suggesting that the influence of COVID-19 on this industry was pronounced in highly populated areas. In contrast, Fukui, Kochi, and Kumamoto prefectures had values of $N_{12}/N_6$ close to 1, which means that $N_{12}$ is similar to $N_6$ in these prefectures.

We compared the values of $N_6/N_1$ and $N_{12}/N_6$ for three service industry-related codes: P (services (beauty and fashion)), Q (services (living)), and R (various services such as advertising, telephone, and garbage collection). For industry codes P and R, the values of $N_{12}/N_6$ were lower than $N_6/N_1$ in Tokyo and Osaka. However, $N_{12}/N_6$ for industry Q was not as low as $N_6/N_1$ in these areas.

For industry code S (sports and leisure), $N_{12}/N_6$ was lower than $N_6/N_1$ in Tokyo and Osaka, where this tendency was particularly significant for Osaka. Industrial category S includes various subcategories associated with amusement: “facilities such as sports clubs, gyms, and dojos;” “pachinko, slot machines, and game centers;” “karaoke, bowling, game centers, discos, etc.;” “movie theaters, theaters, and halls;” “fitness clubs and fitness gyms;” and “stores related to boat racing, bicycle racing, horse racing, and auto racing.” Regarding the travel industry, $N_{12}/N_6$ for industry code T (travel) was also smaller than $N_6/N_1$ in Tokyo and Osaka.

In the case of industry code U (education and culture), $N_{12}/N_6$ was significantly lower than $N_6/N_1$ in Osaka, Tokyo, and Nagasaki, where the tendency was prominent for Osaka. For industry X (Finance, publishing and infrastructure), we also observed a tendency where the values of $N_{12}/N_6$ were lower than those of $N_6/N_1$ in Ibaraki and Osaka.

## Discussion

We investigated the change in the number of stores/facilities recorded in the Japanese telephone directory before and after the outbreak of COVID-19 in Japan and found a significant decrease in the number of leisure/amusement stores/facilities such as karaoke venues and travel agencies after the outbreak. Such declines were prominent in Ibaraki, Osaka, and Hyogo prefectures, whereas the decline was less apparent in Tochigi. We also found that the number of restaurants and travel agencies significantly decreased after the outbreak in urban areas such as Osaka.

Our approach enabled us to capture the influence of the COVID-19 outbreak on stores/facilities that could be considered closed spaces, crowded places, or close-contact settings, the so-called three Cs referred to as conditions to be avoided in the government policy for reducing the spread of the virus. We showed that the increase rate $N_{12}/N_6$ of the number of facilities in category U (education and culture) after the outbreak is lower than $N_6/N_1$, the rate before the outbreak, in urban areas like Tokyo and Osaka. The previous studies have revealed the effect of school closures on suppressing the spread of viral diseases [8, 9]. In Japan, school closures were widely implemented as a measure to prevent the spread of COVID-19 [10]. Industrial category U includes the subcategories of “after school activities and lessons.” Therefore, a possible reason for the low values of $N_{12}/N_6$ in U is that facilities for these activities and lessons were closed as well as schools, which had likely made their management more difficult. Moreover, to control the concentration of tourists at potentially crowded sightseeing spots, the government’s Go To Travel project, originally designed to promote tourism, was cancelled nationwide from the end of December 2020 to early January 2021. Consistent with the suspension of the project, we found that the ratio $N_t/N_1$ of T671 (travel agencies) decreased in May 2021 in Tokyo and Osaka. Finally, our results showed a significant decrease in the number of karaoke facilities after the outbreak in many prefectures, as the Japanese people generally believe that singing in indoor spaces may increase the risk of infection [11]. In sum, our results would seem to confirm the impact of the COVID-19 outbreak on stores/facilities associated with the three Cs.

Our results also appear to confirm the effect of the subsidies made available for the management of stores/facilities during the pandemic. We show a relatively small temporal decline in the number of stores/facilities in October 2020, particularly for the categories of restaurants, sports and leisure, and travel. This likely reflects the impact of the small business sustainability subsidy issued by the Japan Chamber of Commerce and Industry (JCCI) in August 2020, which was seen as a way of preventing or reducing store bankruptcies and closures.

Our results also captured the influence of the emergency declarations on the management of stores/facilities in Japan. In our detailed examination of temporal changes in the number of stores/facilities $N_t/N_{t-1}$ for S645 (“karaoke, bowling, game centers, discos, etc.”) and T671 (“travel agency”), we observed sharp decreases in the values after March 2021 in Ibaraki, Osaka, Hyogo, and Tokyo prefectures. These results can be partially attributed to a series of government emergency declarations, including an emergency declaration issued in January 2021 in Tokyo, Saitama, and other neighboring prefectures of Ibaraki, and another issued in April 2021 for six cities/towns in Ibaraki reducing the open hours of stores and requesting residents to refrain from going out. A state of emergency was also declared in Osaka and Hyogo in mid-January 2021 and extended through early March. The decreases in $N_t/N_{t-1}$ in our results would seem to reflect the impact of these repeated emergency declarations on stores/facilities and their role in restricting the flow of customers.

Of course, our study is not without limitations. The most significant limitation stems from the fact that the telephone directories that served as our principal data source are unable to precisely show the number of bankruptcies over the period studied. Nevertheless, many of our results based on changes in the number of stores/facilities recorded in the directories can be interpreted as reflecting the impact of COVID-19-related events on stores/facilities management. Our analysis did not include stores/facilities that were not registered in the telephone directory data or those without a landline telephone. In seeking to reduce the bias in our data, we based our analysis on the ratios of the number of stores/facilities recorded in the data rather than on the absolute values. Using these ratios, we evaluated whether the number of stores/facilities recorded after the outbreak was significantly lower than the number recorded before the outbreak, under conditions in which both records should be edited with similar biases. We also confirmed the frequency of updates of the data by prefecture. Acknowledging that any analysis requires a consideration of the various biases specific to the data, we nevertheless suggest that the impact of the COVID-19 pandemic can be captured by the type of phone book analysis employed in our study.

## Conclusion

The impact of the COVID-19 pandemic on the economy and society has been explored extensively from various industrial perspectives. In this study, we examined changes in the number of stores/facilities registered in the Japanese telephone directory over a period extending from before to after the outbreak of the pandemic. Specifically, we examined the change in the number of stores/facilities by prefecture, industry, and detailed industry. Many of the results were consistent with previous findings regarding the impact of COVID-19 on various industries. Similar to our attempt in this study, it seems possible to evaluate the impact of an epidemic using an approximation based on the number of records found in phone book data.

Future studies may be able to determine the distinctive characteristics of stores/facilities that experience difficulties in their management during a pandemic by analyzing in greater detail information such as the location of closed and newly opened stores/facilities. The results of such an evaluation can then be compared with those from a previous study [12] that visualized stay-at-home levels by prefecture and municipality in order to assess the characteristics of regional pandemic responses.
